# Genetic parameters for faecal egg count, packed-cell volume and body-weight in Santa Inês lambs

**DOI:** 10.1590/S1415-47572009005000032

**Published:** 2009-03-13

**Authors:** Raimundo N. B. Lôbo, Luiz S. Vieira, Amaury A. de Oliveira, Evandro N. Muniz, José M. da Silva

**Affiliations:** 1 Embrapa Embrapa Caprinos e Ovinos, Sobral, CEBrazil; 2 Embrapa Tabuleiros Costeiros, Aracaju, SEBrazil

**Keywords:** genetic correlations, heritability, Legendre polynomials, random regression

## Abstract

Worm infection is one of the main factors responsible for economic losses in sheep breeding in Brazil. Random regression analysis was used to estimate genetic parameters for the factors faecal egg-count (FEC), packed-cell volume (PCV) and body weight (BW) in Santa Inês lambs. Data from 119 female, offspring of nine rams, were collected between December, 2005 and December, 2006, from the experimental flock of Embrapa Tabuleiros Costeiros, the Brazilian Agricultural Research Corporation located in Frei Paulo, SE, Brazil. After weaning, females were drenched until the faecal egg count had dropped to zero. Two natural challenges were undertaken. FEC heritability was extremely variable, this increasing from 0.04 to 0.27 in the first challenge and from 0.01 to 0.52 during the second. PCV heritability peaks were 0.31 and 0.12 in the first and second challenges, respectively. In the second challenge, BW heritability was close to 0.90. The genetic correlations among these traits did not differ from zero. There is the possibility of increasing parasite resistance in Santa Inês by selecting those animals with lower FEC. Selection to increase resistance will not adversely affect lamb-growth, although lambs with a slow growth-rate may be more susceptible to infection.

## Introduction

In Brazil, worm infection is one of the main causes of economic losses in sheep raising. In this country, gastrointestinal nematode parasitism is controlled basically by means of anthelmintics (Melo *et al.*, 2003). Consequently, and as occurs world-wide, the increasing prevalence of anthelmintic resistance is a fact (Amarante *et al.*, 1992; Echevarria *et al.*, 1996; Melo *et al.*, 2003, Thomaz-Soccol *et al.*, 2004).

Efficient worm control is only possible through the integration of several specific methods, such as grazing management, nutritional supplementation, the strategic use of anthelmintics, vaccines and predatory fungi, as well as breeding for resistance, all contained in a protection program (CSIRO, 2007). The existence of genetic variation among individuals as regards susceptibility to parasitism has been investigated for several years. Genetic differences, both among breeds (Costa *et al.*, 1986; Bricarello *et al.*, 2004; Amarante *et al.*, 2004) and within breeds, have been reported. The most commonly used indicator of host resistance to parasites in sheep is the faecal egg count (Pollot *et al.*, 2004).

Knowledge on genetic parameters of related traits is essential for establishing a resistance breeding program. Among these, trait heritability is one of most important properties. This expresses the proportion of total variance that is attributable to differences in breeding values, and assumes a predictive role, expressing the reliability of phenotypic value as a guide to breeding value (Falconer and Mackay, 1996). The heritability of a trait indicates whether there is the possibility of obtaining genetic gain through its selection. Studies have reported estimates from 0.00 to 0.70 for faecal egg count heritability (Pollot *et al.*, 2004; Vanimisetti *et al.*, 2004; Bishop *et al.*, 2004). Thus, the selection of animals with the lowest faecal egg counts is a means of promoting an increase in host resistance to parasites in sheep. The aim of this study was to investigate genetic parameters for faecal egg count (FEC), packed cell volume (PCV) and body weight (BW) in female Santa Inês hair-sheep lambs, by random regression data analysis.

**Figure 1:**
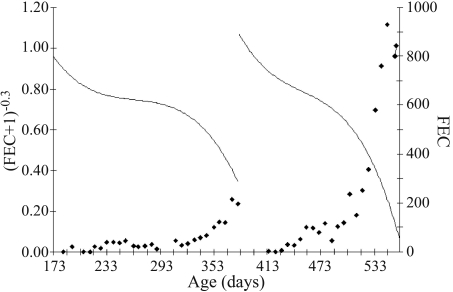
Average fixed curve of transformed FEC estimated by a random regression model for the two challenges (plotted points are FEC non-adjusted raw FEC).

**Figure 2:**
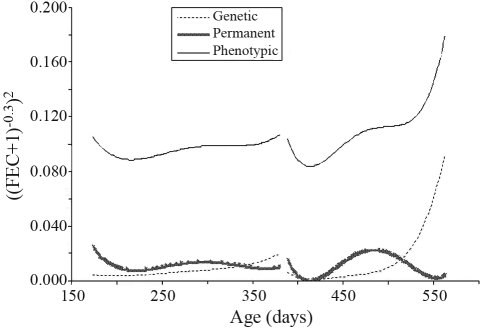
Estimates of phenotypic, direct genetic and direct permanent environmental variances for transformed FEC estimated by a random regression model for the two challenges.

**Figure 3:**
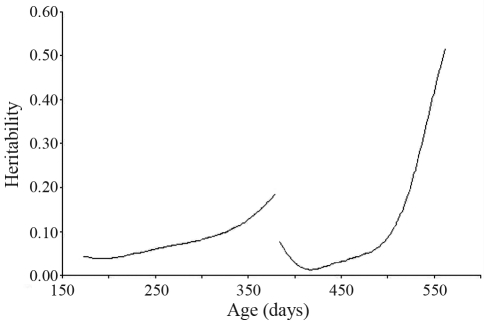
Heritability of transformed faecal egg count (FEC) estimated by a random regression model for the two challenges.

**Figure 4:**
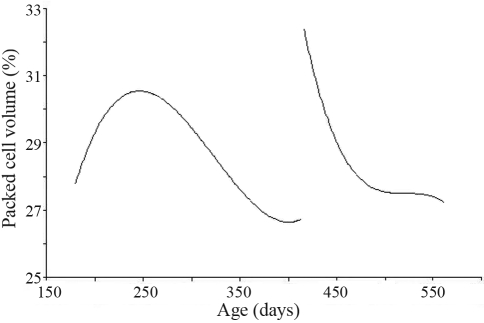
The average fixed curve of packed cell volume estimated by a random regression model for the two challenges.

**Figure 5:**
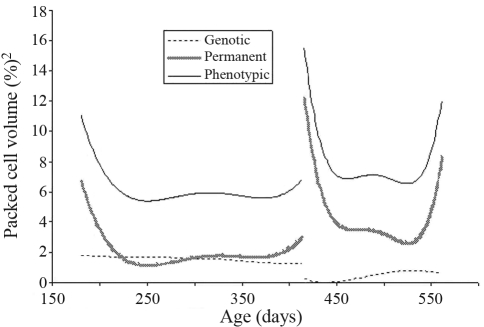
Estimates of phenotypic, direct genetic and direct permanent environmental variances for packed cell volume estimated by a random regression model for the two challenges.

**Figure 6:**
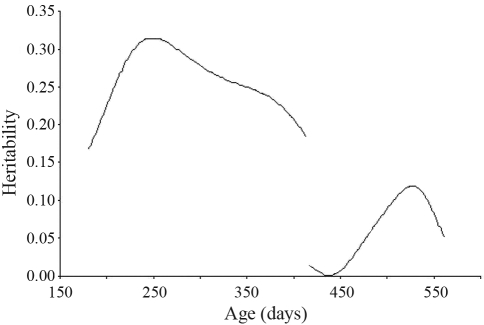
Heritability of packed cell volume estimated by a random regression model for the two challenges.

**Figure 7:**
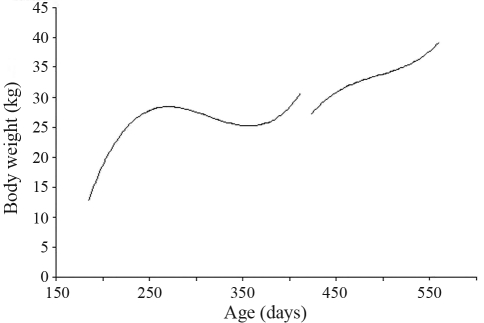
The average fixed curve of body weight estimated by a random regression model for the two challenges.

**Figure 8:**
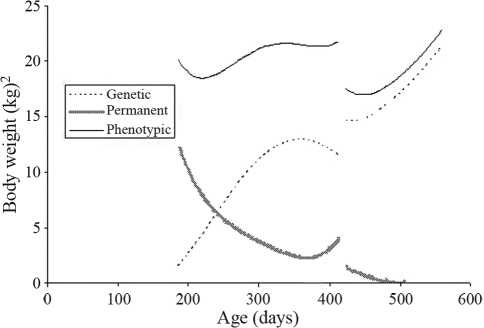
Estimates of phenotypic, direct genetic and direct permanent environmental variances for body weight estimated by a random regression model for the two challenges.

**Figure 9:**
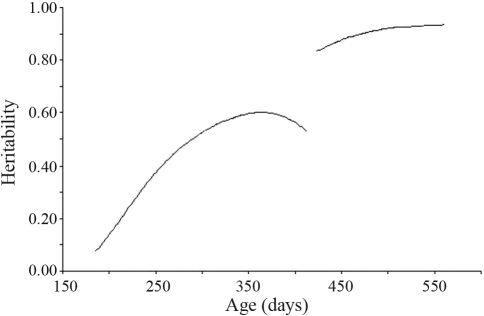
Heritability of body weight estimated by a random regression model for the two challenges.

**Figure 10:**
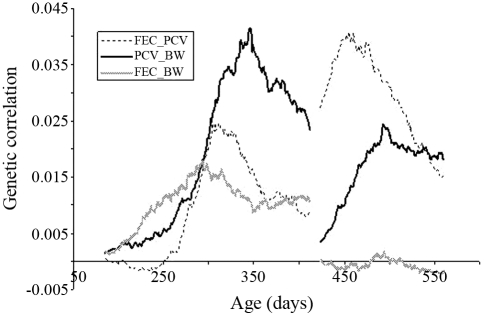
Genetic correlations between faecal egg count (FEC) and packed cell volume (PCV) and body weight (BW), and between PCV and BW estimated by correlations of animal breeding values within age-groups for the two challenges.

## Material and Methods

### Flock data

Data were collected between December, 2005 and December, 2006 from 119 females, the offspring of nine rams (minimum of 11 and maximum 14 offspring per ram), in an experimental flock of Embrapa Tabuleiros Costeiros, the Brazilian Agricultural Research Corporation in Frei Paulo, SE, Brazil. The municipality of Frei Paulo is located at 10° 32' 58" S and 37° 32' 04" W, at an altitude of 272 m. The climate is hot tropical and semi-humid (Köppen, 1931), with temperatures up to 30 °C. The flock is located in a transitional region between the coastline and the semi-arid area of Northeast Brazil. The rainfall in the period was 1,513 mm, and was concentrated between March and August.

There were 107 dams, grouped into the following age-classes: 1) from 1.9 years to 2.2 years old (17 dams); 2) from 2.9 years to 3.05 years old (17 dams); 3) from 4.6 years to 5.65 years old (35 dams); 4) from 5.7 years to 6.10 years old (21 dams); and 5) up to 6.10 years old (17 dams). These age classes were determined after verifying distribution of ages of the ewes.

232 animals constituted the pedigree structure, this consisting of 9 rams (minimum of 11 and maximum of 14 offspring per ram), 107 dams and 116 lambs. Purebred Santa Inês hair-sheep were raised on native pasture (“caatinga”), with supplementation during dry periods. The contemporary offspring were born between June 6 and July 16. Birth types were single, twin and triplet. After weaning (November 2005), all female lambs were drenched until the faecal egg-count (FEC) had been reduced to zero, prior to separation for management on contaminated pastures. When the average FEC reached more than 800 eggs per gram, all the animals were subjected to salvage anthelmintic treatments. Anthelmintic treatments used to clear up parasitic infection before the two challenges are presented in Table 1. Subsequently, in July, 2006, the animals underwent a second challenge (drenched until FEC reached zero, followed by management in contaminated pastures). These challenges were undertaken according to McEwan (1994).

During the experiment, FEC, packed cell volume (PCV) and body weight (BW) were measured every fortnight. Faecal egg counts were determined by using the modified McMaster technique (Gordon and Whitlock, 1939), in which each nematode egg counted represented 100 eggs per gram of faeces. Blood samples were collected by jugular vein puncture into vacutainer tubes containing EDTA. Packed cell volumes were determined by micro-hematocrit centrifugation.

### Statistical analysis

The faecal egg count (FEC) was adjusted through (FEC+1)^-0.3^ transformation, so as to normalize data. This process was not necessary for either PCV or BW. The traits FEC, packed-cell volume and body-weight were analyzed by adjusting random regression models to each challenge, according to the general model:


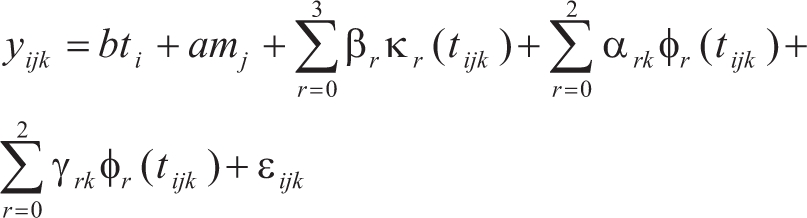


where *y*_*ijk*_ is a record taken at age *t*_*ijk*_ for animal *k* born in birth type -*i*, whose dam belongs to the age-class *j*.  β_*r*_ are the coefficients of fixed cubic regression modeling mean age trends.  Covariables κ_*r*_(*t*_*ijk*_) are the ordinary *r*^-^^*th*^ polynomials evaluated for *t*_*ijk*_.  φ_*r*_(*t*_*ijk*_) are the Legendre *r*^-^^*th*^ polynomials, evaluated for *t*_*ijk*_.  α_*rk*_ and γ_*rk*_ denote the *r*^-^^*th*^ random regression coefficient for direct additive genetic and permanent environmental effects of animal *k*.  Finally, ε_*ijk*_ is the residual error for each *y*_*ijk*_.

The same model was utilized for all traits after better data adjusting had been determined by preliminary analysis. The Legendre polynomial was also tested in order to adjust the fixed curve. B-spline functions were tested for random effects. The criteria used for choosing the better adjustment were: Logarithm of Restricted Maximum Likelihood, Akaike Information Criteria (AIC) and the Schwarz Bayesian Information Criteria (BIC).

The analyses were accomplished by using WOMBAT software (Meyer, 2006).

The relationships between data to determine phenotypic correlations among transformed FEC, PCV and BW were determined by using Pearson's correlation. According to Van Vleck *et al.* (1987), correlationships among breeding values may be considered as the proper definition of genetic correlations. Thus, to obtain these from the analyzed traits, Pearson's correlations were applied to the estimated breeding values of the traits within age-groups.

## Results

Figure 1 presents the average fixed curve of transformed FEC adjusted by ordinary cubic polynomials to the two challenges. 3382 and 2420 records of FEC for the first and second challenges, respectively, were analyzed with a corresponding transformed average of 0.707 and 0.705, for the periods 173 to 383 and 412 to 562 days old. On a real scale these averages correspond, respectively, to 2.176 eggs/g faeces and 2.206 eggs/g faeces. Since the transformed data were on inverse scale as against real data, a consequential increase in FEC was observed in the two challenges. This increase was greater in the second than in the first challenge, probably due to coincidence with the rainy season, and most probably contributing to the trend of the curve.

Estimates of variance components for FEC analysis, when adjusting Legendre polynomials as base functions, are presented in Figure 2. The increase in phenotypic variance was more pronounced in the second challenge. In the first this was practically constant. A similar trend was observed for direct genetic variance. There was a similarity in the shape of the direct permanent environmental variance curve between the two challenges, although this trend was smoother in the first.

Estimates of heritability for FEC were extremely variable (Figure 3). As the animals underwent pasture-contamination during the first challenge, heritability increased from 0.04 to 0.27. In the second challenge, this variation was more pronounced, with a slight decrease in the beginning, but with a subsequent intense increase from 0.01 to 0.52.

Figure 4 shows the average fixed curves of packed cell volume (PCV), adjusted by ordinary cubic polynomials, in both challenges. The curves were very distinct one from the other. In the first, this was sigmoid-shaped, whereas in second there was a diminishing trend.

Variance component estimates for packed cell volumes in both challenges, adjusted by means of Legendre polynomials as basic functions, are presented in Figure 5. There was a certain similarity between the shapes of the curves of phenotypic variance for both packed cell volume and transformed FEC in the two challenges. This suggests that the variation of packed cell volume was the inverse of that for real FEC. As FEC increased there was a reduction in PCV. PCV direct genetic variance slowly declined during the two challenges. On the contrary, the trend of direct permanent environmental variance was similar to that of phenotypic variance.

There were appreciable differences in the heritability estimates for PCV in the two challenges (Figure 6). The heritability peak in the first challenge was 0.31 whereas in the second this was only 0.12.

Figure 7 presents the growth curve of females during the studied period. The animals' weights increased in both challenges.

Phenotypic and direct genetic variances in body-weight increased during the period (Figure 8), whereas the permanent environmental variance for this trait decreased. The levels of genetic variation were higher in the second challenge, although there were lower permanent environmental variations. These aspects were confirmed through high heritabilities for body weight in second challenges (Figure 9). These heritabilities were close to 0.90, thus indicating certain overestimation in these estimates.

Considering the two challenges, the phenotypic correlations between transformed FEC with both PCV and BW were 0.28 (p < 0.0001) and 0.04 (p = 0.0246), respectively. This same correlationship between PCV and BW was 0.03 (p = 0.0831). The genetic correlations between FEC and both PCV and body weight and between PCV and body weight, for the two challenges, is shown in Figure 10. The observed variation of correlations did not differ from zero. The highest genetic correlations were 0.04, 0.04 and 0.02, respectively, between FEC and PCV, body weight and PCV, and FEC and BW. The lowest correlation was observed between FEC and BW (-0.002).

## Discussion

Based on results derived from coproculture,100% of the larvae involved were *Haemonchus sp.*. Furthermore, according to epidemiologic studies in Brazil, sheep raised in the Northeast are infected by *Haemonchus contortus*, *Trichostrongylus axei*, *Trichostrongylus colubriformis*, *Strongyloides papillosus*, *Cooperia* spp., *Oesophagostomum columbianum*, *Trichuris globulosa* and *Skrjabinema* sp. (Arosemena *et al.*, 1999). *Haemonchus contortus*, *T. colubriformis*, *S. papillosus* and *O. colubianum* are those nematodes with the most elevated prevalence and intensity of infection, thereby being considered as the parasites of the highest economic importance for sheep breeding in this region (Costa and Vieira, 1984).

In this study, FEC increased in both challenges with the contamination of grazing animals, although this increase was higher in the second as a result of continuous re-infection in the contaminated pasture. Vanimisetti *et al.* (2004) observed that FEC increased from 3 to 7 weeks of artificial challenge in weaned lambs, but with annual and seasonal variations.

FEC phenotypic variances were higher in the second challenge, even though levels of non-adjusted raw FEC in the animals were also more elevated (Figure 1; plotted points). This indicates the high-L3 environmental contamination existent in this challenge. FEC genetic variation was also greater during this period. According to Pollot and Greeff (2004), FEC genetic variation in low-FEC environments is high, whereby some rams present genetic predisposition for elevated egg counts even when the challenge is relatively low, *i.e.*, they just have no resistance to the parasites at all.

Pollot *et al.* (2004) reported that there seems to be little permanent environmental effect of FEC on lambs, although the very same genes largely control the trait at different ages. In the present study, FEC permanent environmental variance was also slight.

FEC heritability increased with a rise in infection. Furthermore, it was found to be greater in low and high challenges, but was at a moderate level in intermediate environments (Pollot and Greeff, 2004). At high challenges, some animals have the ability to resist parasites more than others. For these authors, selection schemes could be designed to select against animals that have a high FEC in poor environments, as well as for selecting those that have a low FEC in all environments.

The low heritability apparent in the first challenge was probably due to the tender age of the animals, since until six months old, small ruminants do not respond adequately to gastrointestinal infections. However, as they grow older they gradually become less susceptible to the pathogenic effects of nematodes (Vieira *et al.*, 1997).

Pollot *et al.* (2004) reported that FEC grew with age, from approximately 0.2 at weaning to 0.7 at 400 days, this increase intensifying from approximately 250 days onward. Heritability for a single fecal egg count rises as the lamb matures (Bishop *et al.* 1996).

Vanimisetti *et al.* (2004) estimated heritability for FEC of 0.25, 0.22, 0.20, 0.07 and 0.00, respectively at 3, 4, 5, 6 and 7 weeks after artificial challenge. Weighted average heritabilities for Strongyle and Nematodirus egg counts of 0.26 and 0.38, respectively, were reported by Bishop *et al.* (2004).

In animals raised under open conditions, the first infection (primo infection) which usually occurs after weaning, is permissible, this soon being eliminated by anthelmintic medication at short intervals through anthelmintics from three different chemical groups, whereupon the animals are immediately submitted to the first natural challenge (Stear *et al.*, 1996). This challenge occurs when animals reach around four months old. As the animals are accompanied during two natural challenges, the conclusion of the second evaluation occurs after six months of age, when these already present a certain resistance to worm infection (Vieira *et al.*, 1997). To date, efforts aimed at selecting those animals resistant to worms have been making use of this methodology. The results observed in the present study, where heritability was greater in the second challenge but with high variation in both periods, and not allowing for a definition of the ideal age for beginning selection (maybe between 365 and 550 days), indicate that this should begin at six months of age, at least.

The number of FECs necessary for identifying resistant animals depends on the level of environmental larval infestation. The ideal is that at least two contamination peaks occur during the challenge. Usually, the second peak takes place 17 or 18 weeks after infestation in dry regions as those in semi-arid northeast Brazil. Notwithstanding, there is a peak a short time after the challenge if the level of environmental contamination is high. Personal observation revealed that in irrigated pastures in north Ceará - Brazil, infestation and subsequent mortality had already reached high levels, 30 days after the challenge had begun and with only four FEC. Thus, the criteria to be applied indicates drenching the animal when the average FEC of experimental flocks reaches 800 eggs per gram of faeces (McEwan, 1994). As the rain-fall in the study area is concentrated between March and August, this being the period of the highest levels of environmental contamination, it is considered to be the ideal time for parasitological evaluation of the animals with the aim of selecting those which are resistant (Costa and Vieira, 1984).

A reduction in PCV together with an increase in FEC were observed, probably a result of sanguine spoliation caused by *Haemonchus contortus* (100% of the larvae come by in coprocultures were *Haemonchus sp.*), a haematophagous nematode and one of the most common species to be found in this region (Costa and Vieira, 1984). This issue is highlighted by the adequate nutritional conditions of the animals. The females had access to *Brachiaria sp.* pasture with supplemental feed (80% corn bran and 20% soy bran) in the dry period.

Vanimisetti *et al.* (2004) reported a slight decline in PCV, although in this study there were appreciable variations between challenges. Amarante *et al.* (2004) observed means of PCV for Santa Ines sheep higher than 28%, with a lowest mean value of 25.6%.

Contrary to that observed in this study, Vanimisetti *et al.* (2004) reported that heritability estimates were relatively consistent for PCV during infection, this ranging from 0.29 to 0.49. The differences verified between the two studies were probably justified by the methods used to estimate this factor. The methodology employed herein is more complex through using all available information.

The females in this study were measured after weaning, with growth continuing during natural infestation. This trend was similar to that observed by Vanimisetti *et al.* (2004). Nevertheless, it was possible to note that after 250 days during the first challenge, there was a reduction in growth, this coinciding with an increase in FEC levels. Although there was also a rise in FEC levels during the second challenge, there were considerable weight gains during this period. This suggests an increase in parasite resistance with age. These weights are in accordance with the standard body-weight for the Santa Inês breed.

The higher heritability estimates for BW were coincident with moments of higher FEC. This was contrary to reports by Pollot and Greeff (2004) that body weight possessed higher heritability in environments characterized by lower values in environmental variability, stable heritability in the intermediate range of environments, and then decreasing heritability as the environment improves at the upper extreme. These authors concluded that different genes contribute to body weight under different environmental conditions. Pollot *et al.* (2004) reported that BW heritability increased from zero at weaning to approximately 0.7 at 400 days of age.

The positive phenotypic correlations between transformed FEC and PCV (0.28) and with BW (0.04) confirm that lambs with high FEC were associated with a lower PCV during infestation, and a higher FEC with a lighter BW (Vanimisetti *et al.*, 2004).

In this study, the genetic correlations among studied traits did not differ from zero. For FEC and BW, it is possible to suggest that selection for resistance should not have an unfavorable effect on the growth potential of lambs (Vanimisetti *et al.*, 2004). Under UK conditions, Bishop *et al.* (2004) reported that selection goals which place equal emphasis on live-weight and log-transformed egg counts should be a robust method for improving the growth rate and decreasing the larval parasite challenge. However, Pollot *et al.* (2004) observed that the genetic co-relationship between FEC and BW varied from 0 at weaning to -0.63 at the hogget-age in Merino sheep.

Parasite resistance in Santa Inês hair-sheep could be increased by selecting animals for lower FEC, this not adversely affecting growth in lambs, although those with a low growth potential may be more susceptible to infection.

## Figures and Tables

**Table 1:** Anthelmintic treatment used to clear up parasitic infection before each of the two challenges.

Challenge	Period	Active principe	Dose rate	Number of drenches
Oral	Nov/2005	Closantel^1^	10 mg/kg of host body mass	2
Oral	Nov/Dez/2005	Ivermectin^2^	200 μg/kg of host body mass	2
Oral	Dez/2005	Moxidectin^1^	200 μg/kg of host body mass	2
Parenteral (subcutaneous)	Jul/Aug/2006	Moxidectin^1^	200 μg/kg of host body mass	2
